# Stability Modification of Dye-sensitized Solar Cells by Ruthenium Dyes Embedded on Eggshell Membranes

**DOI:** 10.3390/ma16206654

**Published:** 2023-10-11

**Authors:** Naoki Tanifuji, Takeshi Shimizu, Akihiro Shimizu, Kaho Shimizu, Kizuna Abe, Miki Tanaka, Heng Wang, Hirofumi Yoshikawa

**Affiliations:** 1 Chemistry and Biochemistry Division, Department of Integrated Engineering, National Institute of Technology, Yonago College, 4448 Hikona-cho, Yonago 683-8502, Tottori, Japan; 2 Department of Materials Science, National Institute of Technology, Yonago College, Yonago 683-8502, Tottori, Japank.shimigon@gmail.com (K.S.); s160163r@gmail.com (K.A.);; 3 School of Material and Chemical Engineering, Zhengzhou University of Light Industry, Zhengzhou 450002, China; 4 School of Engineering, Kwansei Gakuin University, 2-1 Gakuen, Sanda 669-1337, Hyogo, Japan

**Keywords:** dye-sensitized solar cell, eggshell membrane, electrostatic interaction

## Abstract

Dye-sensitized solar cells (DSSCs) have been one of the most promising technologies to convert sunlight into electricity repeatedly based on the mechanism that dyes inject/accept electron into the metal oxides/from redox mediator. Specifically, N719 ([RuL_2_(NCS)_2_], L: 4,4′-dicarboxy-2,2′-bipyridine), immobilized on TiO_2_ through the interaction between its ligands (-COO^−^ and -NCS) and the oxygen on the TiO_2_ surface, has been used as a conventional DSSC dye with high voltage. Nevertheless, -NCS ligands have been removed from Ru^2+^ in N719 due to UV irradiation and exchanged with H_2_O or OH^−^ in electrolyte, resulting in voltage drop. In this work, we developed the first DSSC using the N719-adsorbed Eggshell (ESM)-TiO_2_ composite to maintain the immobilization of N719 on TiO_2_ through electrostatic interaction between the protein of ESM and N719. The DSSC using the composite maintained the voltage even after 12 h light irradiation, although the voltage of DSSC without ESM dropped drastically. It means that the ESM contributed to stable photovoltaic performances of DSSCs through the protection of NCS ligands of N719.

## 1. Introduction

**Figure 1:**
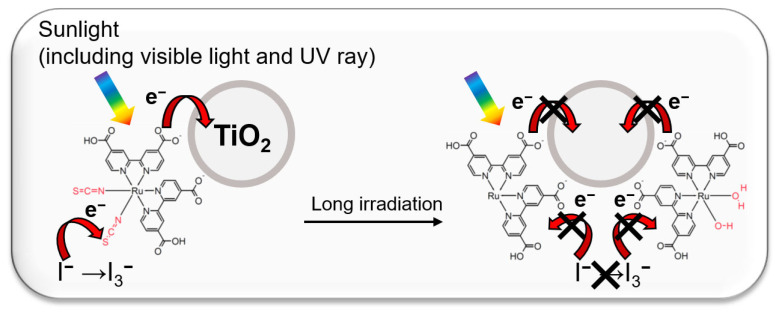
The schematic illustration of the interaction between N719 and TiO_2_ through carboxylate and the surface of TiO_2_, and the conventional DSSC degradation mechanism by long irradiation.

Renewable energies, such as solar, wind, wave and hydro power, have attracted much attention as the solution to increasing energy and environmental concerns [1]. Among them, dye-sensitized solar cells (DSSCs), one of the most promising technologies to convert sunlight into electricity, have been intensely developed due to their lower cost and easier fabrication [2,3]. The typical DSSC, which is composed of a working electrode with dye chemically attached to anatase-TiO_2_, a counter electrode, and electrolyte solution including redox mediator I^−^/I_3_^−^, operates under the following principles [4]: First, the dyes inject electrons into the conduction band of anatase-TiO_2_ upon their excitation by incident light. Second, the injected electrons are transported to the counter electrode through a wire, and then the redox mediator I_3_^−^ is reduced to I^−^ on the counter electrode. Finally, the excited dye then returns to its original state by accepting electrons from the redox mediator I^−^ on the working electrode. As mentioned above, the metal oxide layers, such as anatase-TiO_2_ and ZnO, play an important role in improving the performance of DSSCs by immobilizing dyes through the interaction between the ligands of dyes and oxygen on the metal oxides [5,6,7]. In the conventional DSSCs, N719 ([RuL_2_(NCS)_2_]•2TBA (L = 2,2′-bipyridyl-4-4,4′-dicarboxylic acid; TBA = tetra-*n*-butylammonium) [8,9] have been used as dyes with the ligands (-COO^−^ and -NCS) interacting with oxygen on the surface of TiO_2_ and acting as modulators of the voltages of DSSCs. [10,11,12]. The -COO^−^ group, the major linker to oxygen on TiO_2_ surface, injects electron into the conduction band of TiO_2_ from N719 and elevates the lowest unoccupied molecular orbital (LUMO) energy of N719 [13,14]. The -NCS group, which is the minor linker to the TiO_2_ surface, retrieves electron from a redox mediator and lowers the highest occupied molecular orbital (HOMO) energy [15,16]. This HOMO-LUMO gap modulation determines the absorption wavelength between 300 and 800 nm, resulting in the DSSCs exhibiting a high voltage of 0.7 V [4]. However, the -NCS ligand has been removed from Ru^2+^ in N719 by UV irradiation and exchanged with H_2_O or OH^−^ in electrolyte, resulting in a drop in voltage and photocurrent density [17,18,19,20] (Figure 1). A low-cost and simple solution has also been required for wider application of DSSCs. This suggests that the problem in DSSC technology can be solved by a low-cost system to protect the -NCS ligand of N719.

As a solution to the problem, we focused on the structure of eggshell membrane (ESM). ESM, which has been generally discarded as an unwanted byproduct of egg consumption worldwide, is a waste product with enormous energy potential [21]. ESM has the hydrophobic structure composition of collagen-like proteins with organic substituents, such as hydroxyl, amine and sulfonic groups [22], resulting in ESM being applied to fuel cells as an insoluble proton-conducting membrane through their substituents [21,23,24]. Recently, ESMs have been utilized as low-cost and available biotemplates for the preparation of inorganic nanomaterials [23,24,25,26,27] and adsorbents for the removal of water pollutants [28,29], due to their good adsorption of organic materials and metal ions [30,31] into the hydrophobic cavity. These applications contribute as a simultaneous solution to environmental issues and decrease in waste [30]. Furthermore, it is expected that ESM can serve as a functional material to immobilize dyes in DSSCs, because of its adsorption ability in hydrophobic cavity. As demonstrated in a recent report on solar cells containing biomaterials [32] such as mycobacterial protein (conversion efficiency *η*: 1%) [33], bamboo (*η*: 5.4%) [34], chitosan (*η*: 1.8%) [35] and spinach (retention of max current density after irradiation: 77.78%) [36], the use of biomaterials may be critical to the development of a new class of sustainable and greener solar cells.

**Figure 2:**
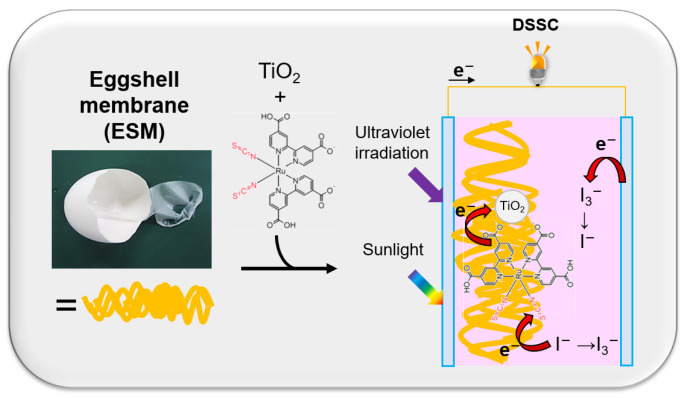
Experimental methodology scheme.

In this work, for the first time, we report the adsorption of N719 ruthenium dyes on ESMs and the results for the application of ESMs to DSSC (Figure 2). We compare the photovoltaic performance and stability to those of a DSSC without ESMs. Based on the results, we discuss the role of ESMs in preventing the degradation of N719 dyes after long irradiation.

## 2. Materials and Methods

### 2.1. Materials

All materials were used without further purification. N719, ethanol, 4-t-butyl phenol, I_2_, LiI and acetonitrile were purchased from Tokyo Chemical Industry Co., Ltd. (Tokyo, Japan). Aqueous titanium oxide precursor solution was obtained from Tokyo Chemical Industry Co., Ltd. (Tokyo, Japan) and Peccell Technologies, Inc. (DSSC experiment kit, PEC-TOM02, Yokohama, Japan). FTO-coated glass slides were purchased from Nishinoda Denko Co., Ltd. (Osaka, Japan).

### 2.2. Preparation

#### 2.2.1. EMS

The inner ESM was obtained from a gently broken commercial chicken egg, washed with distilled water and dried at 25 °C in the air for 24 h. The ESM was ground into powder in an agate mortar.

#### 2.2.2. N719-Adsorbed ESM-TiO_2_ Composite

ESM powder (400 mg) was soaked in a solution of water/ethanol (50:50 vol%), including 1 mg of N719. Then, the residue, N719-adsorbed ESM, was obtained and dried at 25 °C. To achieve a strong interaction between the TiO_2_ and N719-adsorbed ESM, we performed an in situ synthesis of anatase-TiO_2_ by using the sol-gel method [37,38,39]. N719-adsorbed ESM-TiO_2_ composite was obtained by adding N719-adsorbed ESM to the aqueous titanium oxide precursor solution. The composite was pasted onto the FTO substrate several times and heated at 60 °C for a few hours. This substrate was used as the working electrode of the DSSC (the loading area: 1 cm × 1 cm).

### 2.3. Characterization

#### 2.3.1. Scanning Electron Microscopy (SEM) with Energy Dispersion X-ray Spectroscopy (EDX)

SEM was performed on JSM-6610 (JEOL, Tokyo, Japan) to observe the morphology of the samples. The samples (ESM and N719-adsorbed ESM-TiO_2_ composite) on carbon tapes were sputtered with gold in vacuo three times for 2 min. SEM observation was conducted under high vacuum at a voltage of 15 kV and a working distance of 10 mm in secondary electron mode.

EDX was performed to reveal the distribution of the components, N719 and TiO_2_, and absence of eggshell on ESM by calculation of the fluorescence X-ray yield derived from Ru, Ti and Ca. The observation was conducted under the same conditions as those of SEM observations in the EDX with a silicon drift detector (JED-2300, JEOL).

#### 2.3.2. X-ray Diffraction (XRD)

The XRD measurement was performed to confirm the form of TiO_2_ in N719-adsorbed ESM. The XRD pattern of N719-adsorbed ESM was recorded in the 2θ angles of 5–60° on a Rigaku MiniFlex600 (Rigaku, Tokyo, Japan) diffractometer using a Cu-target tube at a voltage of 40 kV, a current of 15 mA and a step scan of 0.02°. The intensities were averaged by calculating four times.

#### 2.3.3. UV-Vis Spectroscopy

Diffuse-reflectance ultraviolet-visible (UV-vis) spectroscopy was performed to confirm the composition of N719-adsorbed ESM-TiO_2_ composite. The spectra of ESM powder, anatase-TiO_2_, N719-adsorbed ESM and N719-adsorbed ESM-TiO_2_ composite were obtained using UV-3600 UV-vis-NIR spectrophotometer (Shimadzu, Kyoto, Japan) in air.

### 2.4. Fabrication of DSSCs

N719-adsorbed ESM-TiO_2_ composite working electrode and the Pt-sputtered counter FTO electrode were placed face-to-face and assembled into **C1**. The electrolyte solution was composed of 0.5 M 4-*t*-butyl phenol, 0.05 M I_2_ and 0.1 M LiI in acetonitrile. For control experiments, we prepared the DSSC-designated **C2** by using the N719-TiO_2_ composite, made by the same in situ sol-gel method without ESM, working electrode (the loading area: 2 cm × 4 cm).

### 2.5. The Photovoltaic Performance Measurement

The photocurrent density (*J*) versus voltage (V) characteristics for **C1** and **C2** were measured before and after 12 h of irradiation by using an IVP-2010 (ASAHI Spectra, Tokyo, Japan) under simulated sunlight from a solar simulator (irradiation unevenness of location: <±2%; HAL-320W, ASAHI Spectra). The light intensity, AM1.5 (100 mW cm^−2^), was calibrated with a pyranometer (H9958-50; ASAHI Spectra).

## 3. Results and Discussion

**Figure 3:**
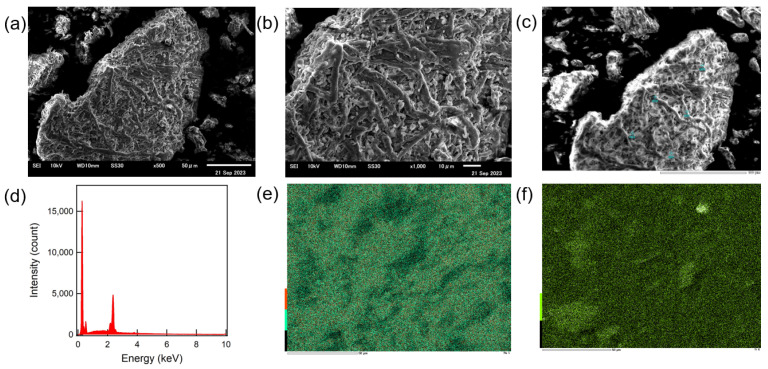
SEM images of ESM powder, magnification of 500 (**a**) and 1000 (**b**). Elemental analysis area of Ca in ESM (five light blue points 001, 002, 003, 004, and 005; (**c**) EDX spectrum at the position 001 (**d**). EDX mapping of the N719-adsorbed ESM-TiO_2_ composite; Ru (**e**) and Ti (**f**).

**Figure 4:**
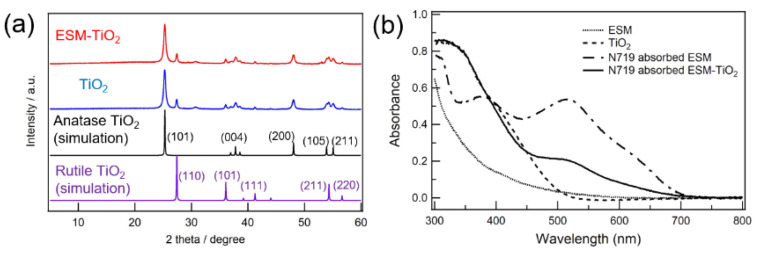
(**a**) XRD patterns of the ESM-TiO_2_ composite, TiO_2_, and simulated data of TiO_2_ (database identifier: ICSD 9852 (anatase), ICSD 23697 (rutile)). (**b**) UV-vis spectra for ESM, TiO_2_, the N719-adsorbed ESM and the N719-adsorbed ESM-TiO_2_ composite.

Figure 3a,b shows an SEM image of the surface of ESM powder. It was revealed that the ESM powder was a spherical aggregate composed of thin fiber. This is because collagen, interwoven and coalescing fiber in ESM was cut by grind in the mortar. Additionally, no Ca ion was detected in the ESM (Figure 3c,d and Figure S1a–d, Table 1, and Tables S1–S4). According to EDX mapping (Figure 3e,f), Ru and Ti were homogeneously detected on the surface of N719-adsorbed ESM-TiO_2_ composite, indicating that N719 and TiO_2_ were distributed on the surface of the ESM fibers homogeneously. Moreover, Figure 4a shows an XRD pattern of the N719-adsorbed ESM-TiO_2_ composite. The peaks were assigned to (101), (004), (200), (105) and (211) of anatase TiO_2_ (quasi-stable phase) [38], although a part of TiO_2_ forms a rutile-form (most stable phase). In addition, a strong absorption occurred at 325 and 525 nm in the UV-Vis spectroscopic observation of N719-absorbed ESM-TiO_2_ composite (Figure 4b). These absorption peaks were assigned as superposition of TiO_2_ and the phenyl groups of their amino acids in ESM (300 nm), N719 (380, 525 nm) and TiO_2_ (325 nm). Therefore, the N719-adsorbed ESM-TiO_2_ composite was composed of the homogeneous EMS, N719 and TiO_2_, and it was evident that TiO_2_ was prepared through the in situ method without decay of N719.

**Figure 5:**
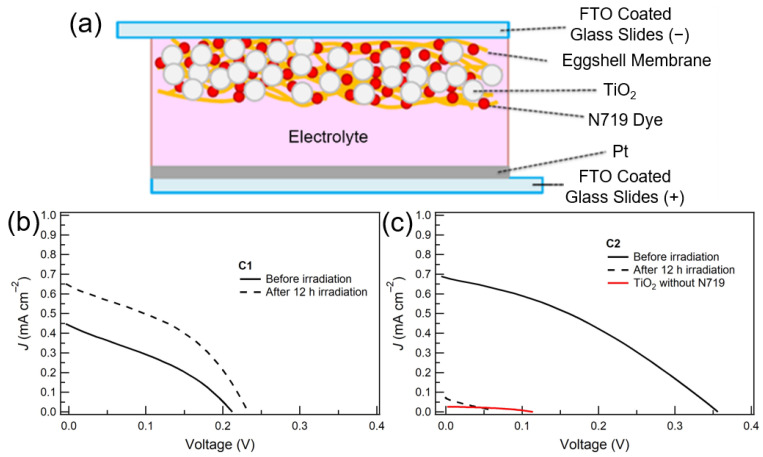
(**a**) Schematic view of **C1**. *J*-*V* curves for the as-prepared **C1** (**b**) and **C2** (**c**) before and after 12 h of light irradiation.

The photovoltaic performances of DSSCs were evaluated using the illustrated structure in Figure 5a, and the *J*-*V* curves of the **C1** and **C2** were obtained before and after 12 h irradiation, respectively (Figure 5b,c). Although **C1** exhibited lower photocurrent densities and voltages than **C2**, before irradiation, the photovoltaic performances of **C1** were higher than those of **C2** after 12 h irradiation. The activation, the gradual resistance-decrease process, might occur in **C1** during irradiation [40], enhancing the photovoltaic performances after 12 h irradiation in **C1**. In addition, Table 2 and Table 3 show the photovoltaic performances, short-circuit photocurrent densities *J*_SC_, open-circuit voltages *V*_OC_, fill factors (*FF*) and power conversion efficiencies (*PCE*) of **C1** and **C2** before and after irradiation, respectively. Before irradiation, **C1** and **C2** showed a *J*_SC_ of 0.441 and 0.682 mA cm^−2^, a *V*_OC_ of 0.212 and 0.356 V, a *FF* of 0.337 and 0.347 and a *PCE* of 0.005 and 0.10%, respectively. On the other hand, after irradiation, **C1** and **C2** showed a *J*_SC_ of 0.643 and 0.071 mA cm^−2^, a *V*_OC_ of 0.232 and 0.063 V, a *FF* of 0.347 and 0.223 and a *PCE* of 0.008 and 0.001%, respectively. It was revealed that ESM-composition improved the photovoltaic stability, although the photocurrent densities and voltages were lower.

**Figure 6:**
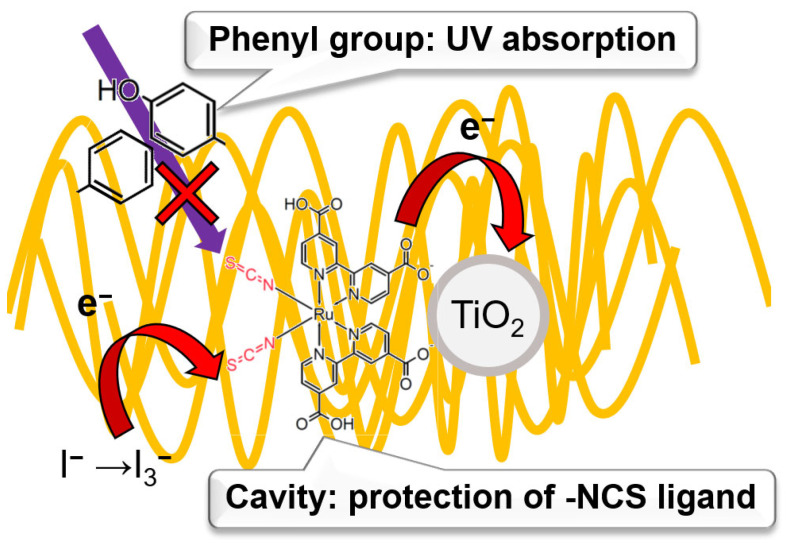
Schematic illustration of the N719-protection mechanism of ESM.

It is assumed that the enhanced stability of **C1** was due to the protection of the -NSC ligand of N719 dyes in ESMs’ hydrophobic cavity. As shown in Figure 6, conventional N719, bound to the surface of TiO_2_ through the interaction between carboxylate and oxygen, has lost -NCS ligands by irradiation, resulting in a voltage drop in **C2**. In addition, photocurrent density was also lowered because of loss of the electron acceptor from the redox mediator I^−^ in electrolyte through the redox reaction of I^−^/I_3_^−^. On the other hand, in this work, -NCS ligand remained because it was surrounded by the ESMs’ cavity [41,42]. Additionally, UV light with a wavelength of 300 nm was absorbed in the phenyl groups of ESMs’ amino acids, such as Phe and Tyr [43] (Figure 4b), maintaining the molecular structure of N719 and the photovoltaic performances in **C1**. As shown in Figure 3b, N719-ESM could accept electron from the redox mediator I^−^ even after irradiation, which likely enhanced the stability of photovoltaic performances of DSSCs.

## 4. Conclusions

We developed a prototypical DSSC using N719-adsorbed ESM-TiO_2_ composites as a working electrode, which is the first example of a DSSC containing ESM. We showed that the new DSSC exhibited more stable performance than a conventional DSSC without ESM. This stability was caused by protection of the dye by ESMs’ structure with the hydrophobic cavity. The present findings thus suggest a novel and effective application for the widely available ESMs, which in the past have mainly been used for the adsorption of chemicals such as organic dyes. However, the photovoltaic performances were significantly lower than those in previously reported DSSCs (Table S5) [2,3,33,34,35], which was likely caused by contamination of rutile TiO_2_, insulating ESM and a small amount of N719 in the electrodes. To improve the values, the DSSC should be optimized by using pure anatase TiO_2_, electronic conductive porous materials and a large amount of N719.

## Figures and Tables

**Table 1:** The elemental analysis at the 001 point.

Element	Energy [keV]	Atomic Ratio [%]
C	0.277	72.87
N	0.392	13.88
O	0.525	9.18
S	2.307	3.10
Mo	2.293	0.60
Au	2.121	0.38

**Table 2:** The performances (mean values) of **C1** and **C2** before irradiation.

DSSC	Short-Circuit Current Density *J*_SC_ [mA cm^−2^]	Open Circuit Voltage *V*_OC_ [V]	Max Power Density *P*_max_ [mW cm^−2^]	*FF*	*PCE* [%]
**C1**	0.441	0.212	0.032	0.337	0.005
**C2**	0.682	0.356	0.085	0.401	0.100

**Table 3:** The performances (mean values) of **C1** and **C2** after 12 h irradiation.

DSSC	Short-Circuit Current Density *J*_SC_ [mA cm^−2^]	Open Circuit Voltage *V*_OC_ [V]	Max Power Density *P*_max_ [mW cm^−2^]	*FF*	*PCE* [%]
**C1**	0.643	0.232	0.060	0.347	0.008
**C2**	0.071	0.063	0.001	0.223	0.001
TiO_2_ (Comparison)	0.057	---	0.008	---	0.001

## Data Availability

The data presented in this study are available on request from the corresponding author.

## Supplementary Materials

The following supporting information can be downloaded at: https://www.mdpi.com/article/10.3390/ma16206654/s1, Figure S1: EDX spectra at the position 002 (a), 003 (b), 004 (c), and 005 (d) of ESM; Table S1: Elemental analysis at the position 002 of ESM; Table S2: Elemental analysis at the position 003 of ESM; Table S3: Elemental analysis at the position 004 of ESM; Table S4: Elemental analysis at the position 005 of ESM; Table S5: The comparison among the conversion efficiencies of DSSCs.
