# Supplementary material for: Stability Modification of Dye-sensitized Solar Cells by Ruthenium Dyes Embedded on Eggshell Membranes
Source: Materials (Basel). 2023 Oct 11;16(20):6654. doi: 10.3390/ma16206654 (PMC10607939; doi:10.3390/ma16206654)
Supplement: Supplementary file 1 [file materials-16-06654-s001.zip › materials-2615791-supplementary.pdf]

materials

Supplementary Materials

Stability Modification of Dye-sensitized Solar Cells by Ruthenium Dyes Embedded on Eggshell Membranes

Naoki Tanifuji *, Takeshi Shimizu*, Akihiro Shimizu, Kaho Shimizu, Kizuna Abe, Miki Tanaka, Heng Wang and Hirofumi Yoshikawa *

[1] Chemistry and Biochemistry Division, Department of Integrated Engineering, National Institute of Technology, Yonago College, 4448 Hikona-cho, Yonago 683-8502, Tottori, Japan

[2] Department of Materials Science, National Institute of Technology, Yonago College, Yonago 683-8502, Tottori, Japan; k.shimigon@gmail.com (K.S.); s160163r@gmail.com (K.A.)

[3] School of Material and Chemical Engineering, Zhengzhou University of Light Industry, Zhengzhou 450002, China

[4] School of Engineering, Kwansei Gakuin University, 2-1 Gakuen, Sanda 669-1337, Hyogo, Japan

* Correspondence: tanifuji@yonago-k.ac.jp (N.T.); t-shimizu@yonago-k.ac.jp (T.S.); yoshikawah@kwansei.ac.jp (H.Y.)

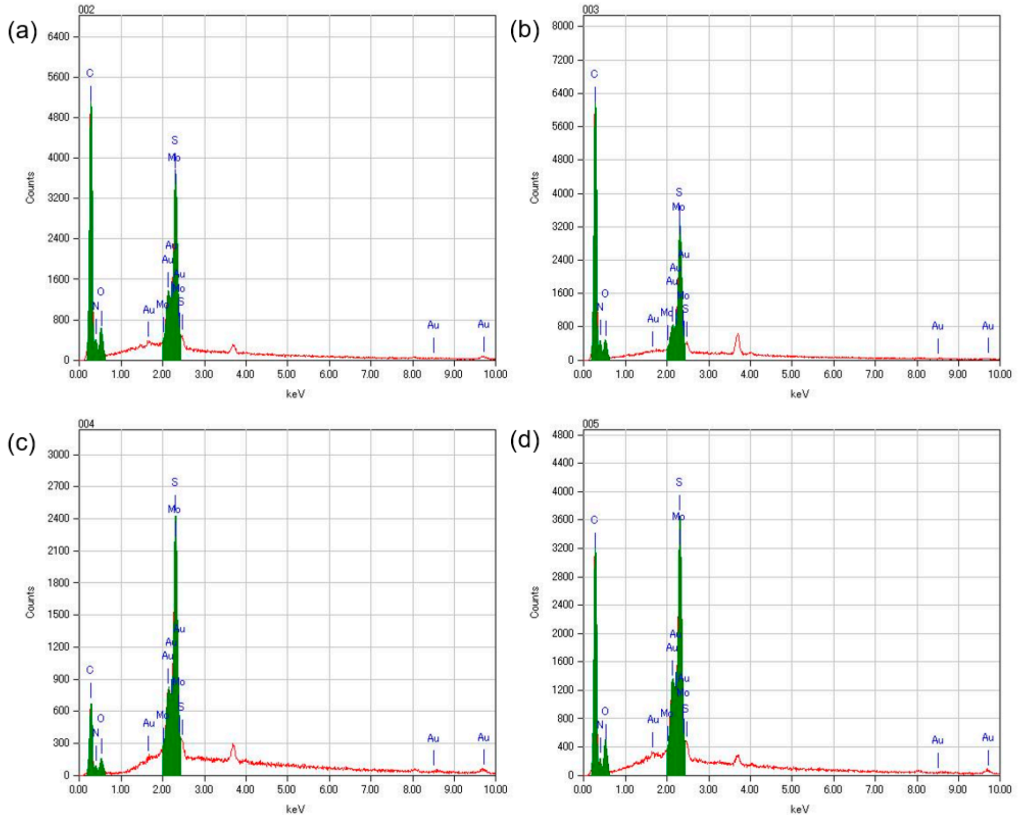

Figure S1. EDX spectra at the position 002 (**a**), 003 (**b**), 004 (**c**), and 005 (**d**) of ESM.

Table S1. Elemental analysis at the position 002 of ESM.

Element	Energy [keV]	Atomic ratio [%]
C	0.277	72.82
N	0.392	12.96
O	0.525	7.22
S	2.307	5.23
Mo	2.293	0.96
Au	2.121	0.81

Table S2. Elemental analysis at the position 003 of ESM.

Element	Energy [keV]	Atomic ratio [%]
C	0.277	72.10
N	0.392	16.56
O	0.525	6.02
S	2.307	4.26
Mo	2.293	0.67
Au	2.121	0.40

Table S3. Elemental analysis at the position 004 of ESM.

Element	Energy [keV]	Atomic Ratio [%]
C	0.277	67.60
N	0.392	9.00
O	0.525	5.42
S	2.307	13.71
Mo	2.293	2.46
Au	2.121	1.80

Table S4. Elemental analysis at the position 005 of ESM.

Element	Energy [keV]	Atomic Ratio [%]
C	0.277	76.85
N	0.392	6.69
O	0.525	7.18
S	2.307	7.17
Mo	2.293	1.03
Au	2.121	1.07

Table S5. The comparison among the conversion efficiencies of DSSCs.

Dye ∣ Metal Oxide	Conversion Efficiency [%]	Reference
$[Ru(bpy)_2(CN)_2]_2Ru(bpy(COO)_2)_2^{2-}$ ∣ TiO_2	7.1-7.9	[1]
N719 ∣ TiO_2	>11	[2]
N719 ∣ TiO_2-bamboo-charcoal-powder	5.4	[3]
Anthocyanin dye ∣ TiO2-WO3	1.8	[4]
N719-adsorbed ESM ∣ TiO_2	0.008	This work

References

1. O'Regan B. and Gratzel M. A low-cost, high-efficiency solar cell based on dye-sensitized colloidal TiO_2 films. *Nature* **1991**, *354*, 737–740.
2. Grätzel, M. Solar energy conversion by dye-sensitized photovoltaic cells. *Inorg. Chem.* **2005**, *44*, 6841–6851.
3. Chou, C. S.; Chen, C. Y.; Lin, S. H.; Lu, W. H.; and Wu, P.; Preparation of TiO_2/bamboo-charcoal-powder composite particles and their applications in dye-sensitized solar cells. *Adv. Powder Technol.* **2015**, *26*, 711–717.
4. Chawla, P.; Srivastava, A.; and Tripathi, M.; Performance of chitosan based polymer electrolyte for natural dye sensitized solar cell. *Environ. Prog. Sustain. Energy* **2019**, *38*, 630–634.
